# Breast Cancer After Reduction Mammoplasty: A Population-Based Analysis of Incidence, Treatment and Screening Patterns

**DOI:** 10.1097/AS9.0000000000000322

**Published:** 2023-08-21

**Authors:** Ashley E. Drohan, May Lynn Quan, Dale C. Birdsell, Yuan Xu

**Affiliations:** From the * Department of Surgery, Dalhousie University, Halifax, Nova Scotia, Canada; † Department of Oncology, Cumming School of Medicine, University of Calgary, Alberta, Canada; ‡ Department of Community Health Sciences, Cumming School of Medicine, University of Calgary, Calgary, Alberta, Canada; § Department of Surgery, Cumming School of Medicine, University of Calgary, Calgary, Alberta, Canada.

## Abstract

**Background:**

The risk of breast cancer may be decreased in women who undergo reduction mammoplasty. The purpose of this study was to describe the incidence and treatment of breast cancer after reduction mammoplasty and to better understand the use of breast cancer screening modalities in these patients.

**Methods:**

This population-based retrospective analysis utilized the Discharge Abstract Database held by the Canadian Institute for Health Information and the National Ambulatory Care Reporting System to identify all women aged 20 years or older who underwent reduction mammoplasty in Alberta, Canada. The incidence and treatment of breast cancer were compared among patients who underwent reduction mammoplasty and age-sex-matched controls. Imaging utilization, including the use of mammography, ultrasound, and breast biopsy, was also compared.

**Results:**

Between 2003 and 2007, 8021 patients over 20 years old underwent reduction mammoplasty in Alberta. Patients were followed for an average of 12.6 years. Eighty-nine (1.1%) patients who underwent reduction mammoplasty developed breast cancer after surgery, compared to 453 (1.9%) controls (*P* < 0.0001). Among patients diagnosed with breast cancer, there was no difference in patient and tumor characteristics. Women who underwent reduction mammoplasty were more likely to undergo mastectomy for cancer (41.6% *vs* 1.5%; *P* < 0.0001) and were more likely to undergo mammography (66.7% *vs* 58.7%; *P* < 0.0001), ultrasound (29.2% *vs* 26.2%; *P* < 0.0001) and biopsy for benign disease (7.2% *vs* 6%, *P* < 0.0001) compared to controls.

**Conclusions:**

Despite an increased frequency of breast cancer screening, the incidence of breast cancer is lower after reduction mammoplasty compared with women who did not undergo breast reduction. After a diagnosis of breast cancer, surgical treatment patterns differ between groups, whereby mastectomy is more common after reduction mammoplasty.

## Abstract 2

Mini abstract

This population-based analysis of 8021 women who underwent reduction mammoplasty between 2002 and 2007 demonstrated that after breast reduction, patients are more likely to undergo breast cancer screening and biopsy for benign disease but are less likely to develop breast cancer compared with the general population.

## Introduction

Reduction mammoplasty is a common surgical procedure typically performed to reduce symptoms of macromastia or to establish breast symmetry in patients who have undergone breast cancer surgery. Recently, several large registry trials have demonstrated a reduced incidence of breast cancer after reduction mammoplasty compared with the general population.^1–5^ The mechanism for the association is not well understood but may be related to the lower volume of breast parenchyma after breast reduction.

In addition to differences in breast cancer incidence between women who undergo reduction mammoplasty and the general population, there also may be differences in the detection and treatment of breast cancer between groups. Screening mammography, which starts at age 50 years for average-risk Canadians, on the postsurgical breast may be less sensitive and specific when compared with those who have never undergone breast surgery. Specific concerns related to mammography after reduction mammoplasty include the presence of fat necrosis, nipple elevation, and other skin thickening.^6,7^ Therefore, it is plausible that after reduction mammoplasty, women may have a higher incidence of abnormal screening results and therefore undergo breast imaging more frequently compared with those patients who have never undergone breast surgery. Similarly, the surgical treatment of breast cancer may be influenced by imaging results after a reduction mammogram, whereby abnormal imaging findings in the breast may prompt patients or providers to remove all abnormal breast tissue.

Understanding the screening behavior, incidence, and subsequent treatment of breast cancer after reduction mammoplasty may improve preoperative patient counseling. Therefore, the objective of this study is to describe the real-world impact of reduction mammoplasty on breast cancer diagnosis, incidence, and management.

## METHODS

### Data Source

This provincial population-based retrospective analysis used the Discharge Abstract Database (DAD) and the National Ambulatory Care Reporting System (NACRS) held by the Canadian Institute for Health Information. The DAD is a national database that captures administrative, clinical, and demographic information on hospital separations (admissions, discharges, deaths, sign-outs, and transfers). NACRS contains data for all hospital-based and community-based ambulatory care, including day surgery. Diagnostic and therapeutic records are recorded according to the International Statistical Classification of Diseases and Related Health Problems, Ninth Revision, Canada (ICD-9) and the Canadian Classification of Health Intervention.

Cases of breast cancer were identified using a prospectively maintained, population-based surgical database, Alberta WebSMR, which captures 95% of breast cancer surgeries from 14 institutions in Alberta. Tumor characteristics and surgical treatment variables were abstracted from Alberta WebSMR. Screening behavior (mammography, ultrasound, and breast biopsy) was identified using provincial physician claims data.

### Patient Population

All women aged 20 years or older who underwent reduction mammoplasty in Alberta, Canada, between 2003 and 2007 were included. Procedural codes for reduction mammoplasty included 1.YM.78 from DAD/NACRS and 97.3 from physician claims data. Patients with a previous diagnosis of malignancy were excluded. Using the nearest neighbor method, a comparator group of 3:1 age-sex-matched controls of the general population in Alberta was created to evaluate the association of reduction mammoplasty with our primary and secondary outcomes.

### Outcomes and Covariates

The incidence of breast cancer was compared between our reduction mammoplasty cohort and age-sex-matched population controls. Similarly, we compared the incidence of benign breast disease (ICD-9 codes 217 and 610) between groups. Among those patients who developed breast cancer during follow-up, we performed a subgroup analysis describing patient demographics, tumor characteristics, and surgical treatment in both groups.

We evaluated differences in screening behavior among women who underwent reduction mammoplasty to those who did not. Screening behavior included the utilization of mammography, breast ultrasound, and image-guided breast biopsy at any time during our follow-up period. To include only screening imaging, and exclude any diagnostic studies, we used physician billings for “screening mammography” which includes bilateral imaging as well as unilateral mammography and ultrasound if claimed twice simultaneously. We did not include diagnostic imaging studies to focus specifically on the incidence of abnormal screening imaging which led to further investigation. Breast biopsy included both core-needle biopsy and excisional biopsy. We hypothesized that women who have undergone reduction mammoplasty would have a higher incidence of pathologically benign radiographic abnormalities on postoperative mammograms compared with those without previous reduction, so we, therefore, described the proportion of patients in both groups who underwent breast biopsy that did not yield a diagnosis of breast cancer within 3 months. Collectively, we refer to screening mammography, ultrasound, and biopsy as “screening behavior” throughout this article.

Patient-level covariates included age and Charlson comorbidity index (CCI), a validated comorbidity measure in a wide range of patient populations, including patients with breast cancer.^8^ Length of follow-up was defined as from the date of index reduction mammoplasty to the end of the follow-up period.

### Statistical Analysis

Patient characteristics were described in the entire cohort, as well as in the subgroup that developed breast cancer. Patient characteristics and screening behavior were reported using descriptive statistics, comparing those who underwent reduction mammoplasty to those who did not. Similarly, patient demographics, tumor characteristics, and surgical treatment were compared among those patients who developed breast cancer after reduction mammoplasty to those who developed breast cancer without a history of breast reduction. The χ^2^ test was used to test categorical variables and the Student *t* test for continuous variables. Analyses were performed using SAS statistical software (SAS Institute, Inc., Cary, NC). This study was approved by our institutional research ethics board. All authors have no conflicts of interest.

## RESULTS

This population-based analysis of 32,084 adult women included 8021 women who underwent reduction mammoplasty between April 2003 and March 2007 in Alberta. Their baseline characteristics are outlined in Table 1.

**TABLE 1.** Patient Characteristics Among Women Who Underwent Reduction Mammoplasty Between 2004 and 2007 Compared to Age-sex Matched Controls

	Patients Who Underwent Reduction Mammoplasty (N = 8021)	Age-sex Matched Controls (N = 24,063)	*P* value
Age group, N (%)			1.00
20–30	2131 (26.6)	6393 (26.6)	
31–40	2231 (27.8)	6693 (27.8)	
41–50	2055 (25.6)	6165 (25.6)	
51–60	1208 (15.1)	3624 (15.1)	
61–70	313 (3.9)	939 (3.9)	
71–80	69 (0.9)	207 (0.9)	
>80	14 (0.2)	42 (0.2)	
Year of surgery, N (%)			1.00
2003	1646 (20.5)	4938 (20.5)	
2004	1696 (21.1)	5088 (21.1)	
2005	1534 (19.1)	4602 (19.1)	
2006	1569 (19.6)	4707 (19.1)	
2007	1576 (19.6)	4728 (19.6)	
Length of follow-up (months) Mean (STD)	151.5 (40.3)	149.3 (42)	<0.0001
CCI score, N (%)			<0.0001
0	5603 (69.9)	18,250 (75.8)	
1	1997 (24.9)	4807 (20.0)	
2	301 (3.8)	640 (2.7)	
>2	120 (1.5)	366 (1.5)	
New diagnosis of breast cancer during follow-up, N (%)			<0.0001
Yes	89 (1.1)	453 (1.9)	
No	7932 (98.9)	23,610 (98.1)	

CCI, indicates Charlson comorbidity index.

### Patient Characteristics

Most women underwent reduction surgery between the age of 20 and 50 years, and the number of cases remained relatively stable during each year of the study time period. Patients were followed for an average of 12.5 years. Patients who underwent reduction mammoplasty had higher comorbidity scores compared to population controls (69.9 *vs*. 75.8%; CCI 0; *P* < 0.0001).

### Breast Cancer Incidence

Patients who underwent reduction mammoplasty had a lower incidence of a new breast cancer diagnosis compared to age-sex-matched controls (1.1 *vs* 1.9%; *P* < 0.0001). Patients who underwent reduction mammoplasty were more likely to have human epidermal growth factor receptor 2+ breast cancer (12.4% *vs* 7.7%; *P* = 0.044) and less likely to have metastatic disease at the time of diagnosis (0% *vs* 5.1%; *P* = 0.043) compared to patients with breast cancer who did not have reduction mammoplasty (Supplemental Table 1, http://links.lww.com/AOSO/A242). The surgical treatment of breast cancer also differed between groups, whereby patients who had a prior breast reduction were more likely to undergo a mastectomy (41.6% *vs* 1.5%; *P* < 0.0001).

### Screening behavior

Screening behavior after reduction mammoplasty differs from that of the general population (Supplemental Table 2, http://links.lww.com/AOSO/A243). In our cohort, patients were more likely to undergo a screening mammogram at any time during follow-up if they had undergone breast reduction, compared with those who did not (66.7 *vs* 58.7%; *P* < 0.0001). Similarly, the annual frequency of screening mammograms was higher after reduction mammoplasty. The use of screening breast ultrasound was also higher among women who underwent reduction surgery compared with population controls.

The use of invasive procedures, including breast biopsy, occurred at a higher frequency in patients with a history of breast reduction compared with those who did not. The overall incidence of breast biopsy was higher in this population throughout the entire follow-up period (7.9 *vs* 7.1; *P* = 0.022) but was also increased on an annual basis (*P* < 0.0001).

Over 500 patients underwent a false-positive breast biopsy after reduction mammoplasty, meaning the biopsy did not yield a diagnosis of breast cancer. This was significantly higher than those who did not undergo reduction surgery (7.2 *vs* 6%; *P* < 0.0001).

## DISCUSSION

This population-based analysis of 32,084 adult women has demonstrated that the incidence, screening behavior, and treatment of breast cancer after reduction mammoplasty differs from that of the general population. Specifically, patients who have undergone a previous reduction mammoplasty have a lower incidence of breast cancer, more frequent breast imaging, and different breast cancer surgical therapy, compared with women the same age who have not undergone previous breast surgery.

In our study, we have demonstrated a reduced incidence of breast cancer after breast reduction. This association has been previously described in several populations.^1,2,4,5,9^ A large Swedish registry study of over 30,000 women who underwent cosmetic breast reduction surgery demonstrated a 28% risk reduction in the development of breast cancer 7.5 years after reduction mammoplasty.^4^ A long-term follow-up at an average of 16 years further supported these results with a 30% reduction in breast cancer and a similar 30% reduction in breast cancer mortality after reduction mammoplasty.^3^ Similarly, a recent population-based analysis of 637 women who underwent breast reduction surgery between 2003 and 2017 in Austria found that there was a reduction in breast cancer incidence of approximately 82% after breast reduction.^10^

The mechanism for reduction in breast cancer risk is not well understood. Some women undergoing breast cancer surgery may elect to undergo simultaneous contralateral breast reduction for cosmetic reasons. It has been hypothesized that the additional removal of breast tissue with breast reduction may lead to the removal of occult cancers and decrease the incidence of metachronous breast cancer in this patient population.^11^ A recent comparison of pathologic specimens after breast reduction supported this finding, demonstrating a higher incidence of high-risk breast lesions after unilateral reduction for oncologic purposes compared to bilateral reduction for macromastia.^12^

In our study, we excluded any patient with a previous diagnosis of breast cancer, and the observed difference in breast cancer incidence may be secondary to the removal of breast tissue at risk for cancer during reduction mammoplasty. This relationship is supported by a Danish study of 1245 women who underwent breast reduction between 1943 and 1971 that found a reduction in breast cancer after reduction that was greatest 10 or more years after surgery in women who had 600 g or more breast tissue removed.^2^ Other potential explanations of these findings may relate to systematic differences in lifestyle and other cancer risk factors between cohorts. This explanation is supported by the lower rate of other cancers among women who underwent reduction mammoplasty, including cervical, lung, and gastrointestinal malignancies.^13^ Women may engage in healthy postoperative behaviors such as increased physical activity, reduction in body mass index, and quitting smoking. Therefore, the statistically significant difference in breast cancer incidence observed between our cohort of postbreast reduction patients and age-matched controls may be secondary to differences in unmeasured confounders.

Despite the lower incidence of breast cancer in our population of women who underwent reduction, we found a higher rate of postoperative breast imaging. Overall, the data on breast cancer screening behavior post reduction mammoplasty is sparse. Our results differ from a case-control study published in 2011, which found no difference in the incidence of abnormal mammograms, additional breast imaging, or biopsy rate among 87 women who underwent reduction mammoplasty compared with matched controls of patients who underwent reduction mammoplasty.^14^ Similarly, a 2016 retrospective review of 49 patients who underwent oncoplastic reduction mammoplasty found no difference in abnormal mammogram or biopsy rates compared with women who underwent lumpectomy without reduction.^15^ However, both of these studies are limited by a small sample size and a short follow-up time.

Our findings of the increased use and higher frequency of screening mammography post reduction mammography could be explained by the difficulty interpreting radiographic postsurgical changes in the breast. The rearrangement of glandular breast tissue and subsequent scar formation may be interpreted as suspicious lesions requiring further investigation in the form of repeat imaging or tissue sampling.^1^ Similarly, our findings of increased mastectomy rate after breast reduction among patients who developed breast cancer may relate to additional ipsilateral abnormal breast imaging findings, prompting patients and providers to seek out the removal of all abnormal appearing tissue with mastectomy. Although our noted mastectomy rate of 41.6% is higher than the Canadian national average, high mastectomy rates in Alberta have been previously documented, and further investigation into potential variation in practice patterns may be warranted.^16,1^

After the detection of abnormal findings on a screening mammogram, additional workup may include breast biopsy. Our findings of increased breast biopsy rate after breast reduction support this concept. Importantly, we also found that the rate of false-positive biopsy was also significantly higher after breast reduction. Although breast biopsy is a low-risk procedure, it is well documented that many women experience significant anxiety and distress when notified of abnormal mammogram findings and the need for further investigation, including breast biopsy. This anxiety may be associated with a reduction in quality of life that can persist for many years.^17–19^

Our study has limitations. First, our data is now over 10 years old, and mammographic quality may have improved during this time frame. However, it is well documented that breast reduction causes mammographic findings including skin thickening, fibrotic bands behind the areola, cysts, and skin calcifications secondary to manipulation of breast tissue, and these findings are unlikely to change over time.^6^ Second, our findings of increased breast cancer screening, breast biopsy, and lower breast cancer incidence may be secondary to unmeasured confounders related to breast cancer risk, including family history, body mass index, alcohol intake, and hormonal factors. These variables could not be captured in our matching strategy as our administrative data set lacked this level of granularity. Third, our large sample size of over 32,000 patients may generate statistically significant differences in measured outcomes, which may not be clinically significant. For example, our difference in breast cancer incidence between cohorts was highly statistically significant, but the absolute difference in incidence was small. However, this difference in cancer incidence of approximately one percent can result in important clinical improvements when applied to large populations. Finally, this is a descriptive study, which inherently limits our ability to determine the causality of the described relationship and outcomes. Similarly, missing data can be a limitation of population-based studies. Although we have an overall low proportion of patients with missing data, over one-quarter of patients with breast cancer in both cohorts had an unknown human epidermal growth factor receptor 2+ status. This is likely related to pathology reporting during our study time frame.

Despite these limitations, our study has demonstrated important findings that may improve the counseling of patients undergoing reduction mammoplasty. Our study is strengthened by its population-based design, large number of patients, and ability to examine the screening behavior of women after reduction mammoplasty in a real-world setting.

## CONCLUSIONS

This population-based analysis of 32,084 women has demonstrated that the incidence of breast cancer after reduction mammoplasty is lower than in the general population. We have also demonstrated differences in breast cancer treatment and screening. Specifically, women who have undergone breast reduction have a higher frequency of screening mammography and breast ultrasound compared with the general population. Additionally, women who develop breast cancer are more likely to have a mastectomy if they have previously had a reduction mammoplasty. These findings are important for patient counseling before and after reduction mammoplasty.

## Supplementary Material


