# Supplementary material for: Breast Cancer After Reduction Mammoplasty: A Population-Based Analysis of Incidence, Treatment and Screening Patterns
Source: Ann Surg Open. 2023 Aug 21;4(3):e322. doi: 10.1097/AS9.0000000000000322 (PMC10513359; doi:10.1097/AS9.0000000000000322)
Supplement: Supplementary file 1 [file as9-4-e322-s001.pdf]

Supplemental Table 1. Patient, tumor and treatment variables among women who underwent reduction mammoplasty and developed breast cancer compared to age-sex matched controls

	Patients who underwent reduction mammoplasty (N=89)	Age-sex matched controls (N=453)	P- value
Age (mean), N (SD)	55 (10.7)	55 (10.5)	0.933
Age group, N (%)			0.337
<41	9 (10.1)	43 (9.5)	
41-50	23 (25.8)	120 (26.5)	
51-60	26 (29.2)	154 (34)	
61-70	29 (32.6)	106 (23.4)	
71-80	2 (2.2)	24 (5.3)	
>80	0 (0)	6 (1.3)	
Tumor grade, N (%)			0.349
1	11 (12.4)		
2	31 (34.8)	6,306 (26.2)	
3	42 (47.2)	17,757 (73.8)	
Missing	5 (5.6)	43 (9.5)	
Stage at diagnosis, N (%)			0.130
0	12 (13.5)	54 (11.9)	
I	42 (47.2)	180 (39.7)	
II	21 (23.6)	140 (30.9)	
III	13 (14.6)	47 (10.4)	
IV	0 (0%)	23 (5.1)	
Missing	1 (1.1)	9 (2)	
pT stage, N (%)			0.774
T0	12 (13.5)	54 (11.9)	
T1	44 (49.4)	217 (47.9)	
T2	22 (24.7)	132 (29.1)	
T3	8 (9)	26 (5.7)	
T4	2 (2.2)	15 (3.3)	
Missing	1 (1.1)	9 (2)	

pN stage, N (%)			0.223
N0	69 (77.5)	296 (65.3)	
N1	14 (15.7)	111 (24.5)	
N2	3 (3.4)	30 (6.6)	
N3	2 (2.2)	8 (1.8)	
Missing	1 (1.1)	8 (1.8)	
pM stage, N (%)			0.043
M0	88 (98.9)	426 (94)	
M1	0 (0)	23 (5.1)	
Missing	1 (1.1)	4 (0.9)	
ER, N (%)			0.971
Positive	70 (78.7)	351 (77.5)	
Negative	13 (14.6)	70 (15.5)	
Unknown	6 (6.7)	32 (7.1)	
PR, N (%)			0.982
Positive	60 (67.4)	307 (67.8)	
Negative	22 (24.7)	113 (24.9)	
Missing	7 (7.9)	33 (7.3)	
HER-2, N (%)			0.044
Positive	11 (12.4)	35 (7.7)	
Negative	55 (61.8)	242 (53.4)	
Unknown	23 (25.8)	176 (38.9)	
Surgery, N (%)			<0.0001
Lumpectomy	50 (56.2)	419 (92.5)	
Mastectomy	37 (41.6)	7 (1.5)	
None	2 (2.2)	27 (6)	
