# Supplementary material for: Breast Cancer After Reduction Mammoplasty: A Population-Based Analysis of Incidence, Treatment and Screening Patterns
Source: Ann Surg Open. 2023 Aug 21;4(3):e322. doi: 10.1097/AS9.0000000000000322 (PMC10513359; doi:10.1097/AS9.0000000000000322)
Supplement: Supplementary file 2 [file as9-4-e322-s002.pdf]

Supplemental Table 2. Screening behaviour among patients who underwent reduction mammoplasty compared to age-sex matched controls

	Patients who underwent reduction mammoplasty (N=8,021)	Age-sex matched controls (N=24,063)	P- value
Mammography within follow-up period, N (%)			<0.0001
Yes	5,347 (66.7)	14,128 (58.7)	
No	2,674 (33.3)	9,935 (41.3)	
Frequency of mammograms, per year N			<0.0001
(%)	2,674 (33.3)	9,935 (73.8)	
0	2,878 (35.9)	6,305 (26.2)	
0-1	1,550 (19.3)	1 (0)	
1-2	919 (11.5)	0 (0)	
>2			
Ultrasound, within follow-up period N (%)			<0.0001
Yes	2,345 (29.2)	6,306 (26.2)	
No	5,676 (70.8)	17,757 (73.8)	
Frequency of ultrasounds, per year N (%)			<0.0001
0	5,676 (70.8)	17,757 (73.8)	
0-1	2,147 (26.8)	6,305 (26.2)	
1-2	176 (2.2)	1 (0%)	
>2	22 (0.3)	0 (0%)	
Breast biopsy, within follow-up period N (%)			0.022
Yes	633 (7.9)	1,714 (7.1)	
No	7,388 (92.1)	22,349 (92.9)	

Frequency of breast biopsy, per year N (%)			<0.0001
0	7,388 (92.1)	22,349 (92.9)	
0-1	607 (7.6)	1,705 (7.1)	
1-2	23 (0.3)	8 (0)	
>2	3 (0)	1 (0)	
False-positive biopsy, N (%)			<0.0001
Yes	576 (7.2)	1,439 (6)	
No	7,445 (92.8)	22,624 (94)	
